# 3D Printing of Block Copolymer-Based Fracture Tough Denture Base Materials

**DOI:** 10.3390/polym18131660

**Published:** 2026-07-04

**Authors:** Kai Rist, Iris Lamparth, Sadini Omeragic, Lauren Geurds, Benjamin Grob, Yohann Catel

**Affiliations:** Ivoclar Vivadent AG, Bendererstrasse 2, FL-9494 Schaan, Liechtenstein; kai.rist@ivoclar.com (K.R.); iris.lamparth@ivoclar.com (I.L.); sadini.omeragic@ivoclar.com (S.O.); lauren.geurds@ivoclar.com (L.G.); benjamin.grob@ivoclar.com (B.G.)

**Keywords:** denture base, block copolymers, high impact, fracture toughness, 3D printing

## Abstract

The development of 3D printing high-impact denture bases is challenging, as materials exhibiting both high flexural strength/modulus and fracture toughness are required. Nowadays, most of the commercially available 3D printing denture bases contain significant amounts of crosslinking monomers and therefore behave as brittle materials. In this contribution, urethane dimethacrylate **DMA1**/(octahydro-4,7-methano-1H-indenyl)methyl acrylate (OMIMA) 1/1 (wt/wt) formulations containing a poly(ε-caprolactone)-polydimethylsiloxane-poly(ε-caprolactone) (PCL-PDMS-PCL) triblock copolymer (**BCP1**) and fumed silica SiO_2_-NPs were evaluated for DLP 3D printing of fracture-tough denture bases. The post-curing step was performed at various temperatures (RT, 60 °C, 80 °C, 100 °C and 120 °C). This parameter was shown to strongly influence the T_g_ and mechanical properties of 3D printed materials. A post-curing temperature of 100 °C was found to be ideal. Under these conditions, 3D printed materials exhibiting excellent mechanical properties were successfully obtained. Furthermore, the amounts of **BCP1** and SiO_2_-NPs were varied. The formulation containing 8.0 wt% of **BCP1** and 10.0 wt% of SiO_2_-NPs (FS = 67.5 ± 1.3 MPa, FM = 2450 ± 71 MPa, K_max_ = 2.11 ± 0.06 MPa m^1/2^, W_f_ = 1109 ± 19 J m^−2^) was able to fulfill the ISO 20795-1:2013 requirements in terms of flexural strength (FS)/modulus (FM) and fracture toughness for denture bases with improved impact resistance (FS > 65 MPa, FM > 2000 MPa, K_max_ > 1.9 MPa m^1/2^, W_f_ > 900 J m^−2^). This material showed better performance than the commercially available formulations Printodent^®^ GR-14.2 denture HI (FS = 69.2 ± 1.8 MPa, FM = 2153 ± 76 MPa, K_max_ = 0.82 ± 0.04 MPa m^1/2^, W_f_ = 79 ± 10 J m^−2^) and Lucitone Digital Print^TM^ 3D denture base (FS = 56.7 ± 1.9 MPa, FM = 2144 ± 12 MPa, K_max_ = 1.92 ± 0.09 MPa m^1/2^, W_f_ = 1272 ± 177 J m^−2^).

## 1. Introduction

Complete dentures have been traditionally manufactured using compression and injection molding techniques [1]. Heat-cure denture bases are typically obtained via thermal polymerization of a methyl methacrylate (MMA) based resin (small amounts of dimethacrylates are frequently added) [1,2]. The resulting materials mainly consist of PMMA and exhibit a low crosslinking density. They can therefore be efficiently reinforced via the addition of toughening agents such as core–shell particles. Although excellent and clinically successful materials were developed using this procedure, traditional manufacturing of complete dentures remains complex and time-consuming. The development of CAD (computer-aided design)–CAM (computer-aided manufacturing) technology has revolutionized the dental industry. Using this digital approach, denture bases can be prepared either by subtractive (SM) or by additive manufacturing (AM) [3,4,5,6,7,8,9]. Following the SM workflow, pre-polymerized PMMA-based denture bases are milled out of disks using specific milling machines. Although materials exhibiting excellent mechanical properties (flexural strength/modulus, fracture toughness, etc.) are obtained, SM involves significant material waste. On the other hand, AM, also known as 3D printing, produces less waste. It is a fast and cost-effective technology that enables the manufacturing of denture bases with high precision [10,11,12,13,14]. Stereolithography (SLA) and Digital Light Processing (DLP) are mainly used in dental laboratories and practices. Both technologies produce 3D printed materials via the photopolymerization of a resin layer by layer. SLA requires a laser light source to cure the resin, whereas DLP uses a light projector. Various 3D printing denture bases are available on the market. As MMA cannot be efficiently used for 3D printing due to its volatility and low reactivity, the chemical composition of 3D printing denture bases had to be completely modified. They mainly consist of a mixture of dimethacrylates, resulting in printed dentures that are highly crosslinked and brittle.

Denture base materials are subjected to various stresses, such as repeated masticatory forces. High impact forces could also be generated as a result of an accidental dropping of the denture. Such stresses could lead to a fracture of the denture base. For these reasons, fracture-tough denture bases are preferred. Two families of traditionally manufactured denture base materials can be found on the market: conventional and high impact denture bases. The ISO 20795-1:2013 standard [15] for light-activated denture bases does not specify any lower limit regarding fracture toughness. On the other hand, high impact denture bases must exhibit high maximum stress intensity factor (K_max_) and total work of fracture (W_f_) values (K_max_ > 1.9 MPa m^1/2^ and W_f_ > 900 J m^−2^). Geiger et al. as well as Hetzler et al. recently evaluated several commercially available 3D printing denture bases and clearly showed that these materials did not fulfill the requirements for materials with improved impact resistance (K_max_ and W_f_ were significantly lower than the minimum required values) [16,17]. To the best of our knowledge, Lucitone Digital Print^TM^ 3D denture base (Dentsply Sirona) is the only 3D printing denture base material able to exceed the minimum required fracture toughness for high impact materials. However, recent studies reported quite low flexural strength and modulus values using this material [18,19]. There is therefore a strong need for the development of fracture-tough 3D printing denture bases presenting mechanical properties in the same range as the state-of-the-art PMMA-based materials.

According to the state of the art, various technologies were proposed in order to increase the fracture toughness of crosslinked materials. The incorporation of toughening agents such as reactive rubbers (e.g., carboxy-terminated butadiene acrylonitrile copolymers) [20], block copolymers (BCPs) [21], core–shell particles [22], hyperbranched polymers [23], thermoplastic particles [24], silica nanoparticles [25], or polyhedral oligomeric silsesquioxane (POSS) [26] was reported. Although these approaches were shown to be efficient for epoxy materials, most of them were significantly less performant once transferred to crosslinked methacrylate networks [27,28,29].

**Figure 1:**
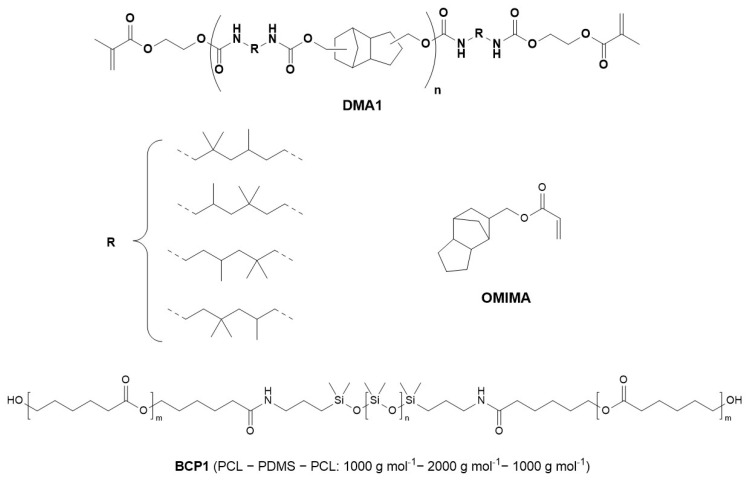
Structures of **DMA1**, **BCP1** and OMIMA.

In the last few years, our research group developed a highly efficient technology to significantly improve the fracture toughness of 3D printable (meth)acrylate-based resins. This technology lies in the incorporation of BCPs in a low crosslinking density network [30,31,32,33]. This network is typically obtained via the photopolymerization of a resin containing a polymerizable urethane macromonomer combined with a monofunctional monomer as diluent. Both diblock and triblock copolymers were evaluated. Amongst the tested toughening agents, poly(ε-caprolactone)-polydimethylsiloxane-poly(ε-caprolactone) (PCL-PDMS-PCL) triblock copolymers were shown to be particularly efficient. Indeed, the addition of **BCP1** (PCL-PDMS-PCL: 1000 g mol^−1^-2000 g mol^−1^-1000 g mol^−1^) to a **DMA1**/OMIMA monomer mixture led to fracture-tough materials exhibiting excellent mechanical properties (Figure 1) [33]. Using an optimized amount of **BCP1**, the ISO 20795-1:2013 standard requirements for high impact denture bases were successfully reached. It is, however, worth mentioning that the materials were photocured in molds.

The objective of this contribution is to evaluate this BCP toughening technology for DLP 3D printing of fracture-tough denture base materials. The **DMA1**/OMIMA/**BCP1** combination was selected for this investigation, as it provided the highest fracture toughness up to now. Several research groups recently reported the strong influence of the post-curing step on the mechanical properties of printed materials [34,35,36,37]. Particularly, it was shown that heating during the post-processing step was advantageous and led to higher flexural strength/modulus values and improved conversion. For this reason, the selected 3D-printed **BCP1**-based resins were post-cured using different conditions (variation in temperature and irradiation time). In this article, the influence of the post-curing conditions on the flexural strength, flexural modulus, and fracture toughness of 3D-printed **BCP1**-based materials is discussed. The difference in mechanical properties between bulk-cured (in a mold) and 3D-printed materials is also addressed.

## 2. Materials and Methods

### 2.1. Materials

The monomer (octahydro-4,7-methano-1H-indenyl)methyl acrylate (OMIMA) was provided by Arkema (Colombes, France) and the initiator Genocure TPO (2,4,6-trimethylbenzoyldiphenylphosphine oxide) by Rahn AG (Zurich, Switzerland). **DMA1** and **BCP1** were synthesized according to previously reported procedures [33]. Fumed silica nanoparticles (SiO_2_-NPs) silanized with 3-methacryloxypropyltrimethoxysilane, and exhibiting a specific surface area (BET) of 25–45 m^2^ g^−1^, were used as received from Evonik (Essen, Germany).

### 2.2. Formulation of Photopolymerizable BCP1-Based Monomer Mixtures

**DMA1** and the monofunctional monomer OMIMA were mixed under magnetic stirring for several hours at 50 °C until full dissolution of the very highly viscous **DMA1**. 1.0 wt% TPO as well as **BCP1** were subsequently added and stirring was continued until full solubilization of **BCP1**. Various **BCP1** amounts were evaluated (4.0 wt%, 5.0 wt%, 6.0 wt%, 7.0 wt% and 8.0 wt%). These values represent the **BCP1** content in the monomer mixture (without taking the filler into account). After addition of SiO_2_-NPs, the material was mixed and homogenized for 2 × 2 min at 2000 U min^−1^ using a SpeedMixer DAC 600.1 (Hauschild SpeedMixer, Hamm, Germany).

### 2.3. Measurement of the Mechanical Properties, Glass-Transition Temperature, and Double Bond Conversion

#### 2.3.1. Bulk Cured BCP1 Containing Materials

##### Flexural Strength and Flexural Modulus

Flexural strength (FS) and flexural modulus (FM) were assessed according to a slightly modified ISO 20795-1:2013 method. Specimens (3.3 mm × 10 mm × 64 mm) were prepared using stainless-steel molds (n = 6). The molds were filled with the photopolymerizable monomer mixture and covered with polyester film (50 µm) to avoid oxygen inhibition. Materials were light-cured in an LED cube 100 (20 mW cm^−2^ @ 405 nm and 100 mW cm^−2^ @ 450 nm, Hönle, Gilching, Germany) controlled by an LED Powerdrive (Hönle, Gilching, Germany) for 10 min for each side. After complete cure, specimens were allowed to cool down to RT, removed from the molds, and sanded from all sides with 600-grit silicon carbide sandpaper. Specimens were then stored in water at 37 °C for 50 h. Measurement of FS/FM was carried out in a three-point bending test (span: 50 mm) with a cross-head speed of 5 mm min^−1^ using a Z2.5/TS universal testing machine (ZwickRoell, Ulm, Germany). The measurement was carried out in water at 37 °C (the specimens were immersed in a tempered water bath during the measurement).

##### Fracture Toughness

Fracture toughness was measured according to a slightly modified ISO 20795-1:2013 method. Both the maximum stress intensity factor (K_max_) and the total work of fracture (W_f_) were determined using single-edge notched beam (SENB) specimens (n = 6). A stainless-steel mold (4 mm × 8 mm × 40 mm) was filled with a photopolymerizable monomer mixture. The mold was covered with a polyester film (50 µm) to avoid oxygen inhibition. Materials were light-cured in an LED cube 100 (20 mW cm^−2^ @ 405 nm and 100 mW cm^−2^ @ 450 nm, Hönle, Gilching, Germany) controlled by an LED Powerdrive (Hönle, Gilching, Germany) for 10 min from both sides. After polymerization of the bulk specimens, a 1.0 mm wide and 3.0 mm deep notch of rectangular shape was prepared using a circular saw with a diamond blade. A pre-crack was then prepared at the bottom of the notch by striking a razor blade with gentle pressure to a depth of 0.3 mm. Specimens were stored in water for seven days at 37 °C, dabbed with paper and subsequently loaded to break at RT with a span of 32 mm and at a crosshead speed of 1.0 mm min^−1^ using a universal testing machine (Zwick Z2.5, ZwickRoell, Ulm, Germany). K_max_ (in MPa m^1/2^) was calculated as follows:

Kmax=f(x)Pmaxlt(btht32)×10−3 MPa m12

where f(x) is a geometrical function dependent on x

f(x)=3x12[1.99−x(1−x)(2.15−3.93x+2.7x2]/[2(1+2x)(1−x)32]

with x = (a/h_t_) and h_t_ as the height of the specimen (8 mm), b_t_ its width (4 mm), l_t_ the span length (32 mm), a the crack length (3 mm + pre-crack depth with razor blade), and P_max_ the maximum load exerted on the specimens (in N).

The total fracture work W_f_ (in J m^−2^) was calculated as follows:

$$W_{f}=\frac{U}{\left[2b_{t}(h_{t}-a)\right]}\times 1000$$

where U is the recorded area under the load/deflection curve.

#### 2.3.2. 3D Printed Materials

##### Commercially Available 3D Printing Denture Base Materials

Two formulations were evaluated: Printodent^®^ GR-14.2 denture HI (pro3dure medical GmbH, Iserlohn, Germany and Lucitone Digital Print^TM^ 3D denture base (Dentsply Sirona, Charlotte, USA). They were printed using the ASIGA UV MAX 385 (Asiga, Alexandria, Australia) with a layer thickness of 100 μm and the printing parameters recommended by the manufacturers. Post-processing was carried out according to the manufacturers’ instructions, which included ultrasonic cleaning in isopropyl alcohol and light exposure in a post-curing device. Printodent^®^ GR-14.2 denture HI was post-processed in an Otoflash G171 unit (NK-Optik GmbH, Baierbrunn-Buchenhain, Germany), whereas Lucitone Digital Print^TM^ 3D denture base was post-cured in a Digital Cure large capacity unit (Dentsply Sirona, Charlotte, NC, USA).

##### **BCP1** Containing Materials: 3D Printing Workflow

All samples were printed to their final dimensions on a DLP printer (PrograPrint PR5, Ivoclar Vivadent AG, Schaan, Liechtenstein) using the parameter set “ProArt Print Splint”. They were printed with a layer thickness of 100 µm and with a light intensity of 16 mW cm^−2^ (λ = 385 nm). The layer exposure time was set to 1.35 s for all mixtures. Specimens were printed without supports and on their thinner side in horizontal orientation (in y-direction) with deactivated burn-in-layers. Excess resin was removed with wipes and specimens were post-cured for 10 min on each side in an LED cube 100 (20 mW cm^−2^ @ 405 nm and 100 mW cm^−2^ @ 450 nm, Hönle) controlled by an LED Powerdrive (Hönle). The specimens were placed on a custom-made pre-heated heating plate with adjustable temperature (set between RT and 120 °C) during the post-curing. After complete cure, the samples were allowed to cool down to RT and sanded from all sides with 600-grit silicon carbide sandpaper, except for the NIR samples, which were measured without sanding.

##### Flexural Strength and Flexural Modulus

Printed specimens (3.3 mm × 10 mm × 64 mm, n = 6) were stored in water at 37 °C for 50 h. The measurement was performed similarly to Section Flexural Strength and Flexural Modulus (for bulk-cured materials).

##### Fracture Toughness

SENB specimens (4.0 mm × 8.0 mm × 40 mm, n = 6) that already contained a 1.0 mm wide and 3.0 mm deep notch of rectangular shape (designed in the STL file) were 3D printed. After printing, the notch was cleaned carefully with paper towels to remove any excess resin, and the specimens were post-cured as described before. A pre-crack was then prepared at the bottom of the notch by striking a razor blade with gentle pressure to a depth of 0.3 mm. Specimens were stored in water for seven days at 37 °C and dabbed. The fracture toughness measurement was performed similarly to Section Fracture Toughness (for bulk-cured materials).

##### Glass-Transition Temperature (DMTA)

DMTA measurements were performed with printed specimens of the dimensions 5 mm × 2 mm × 40 mm on an Anton Paar (Graz, Austria) MCR 301 device with a CTD 600 oven and an SRF 5 fixture. The measurements were carried out in torsion mode with a frequency of 1 Hz, a normal force (FN) of –1 N, and a strain of 0.05%. A temperature spectrum was monitored from 25 °C to 250 °C with a heating rate of 2 K min^−1^. The glass transition temperature (T_g_) was defined as the temperature corresponding to the maximum of the loss factor (tan δ) curve.

##### Double Bond Conversion (NIR Spectrometry)

Spectra of the uncured material (film thickness 1.0 mm) and of printed, circular specimens (d = 15 mm, h = 1.0 mm, n = 3) were measured by NIR spectroscopy using an Invenio R spectrometer (Bruker, Billerica, MA, USA, 16 Scans, 8 cm^−1^ Resolution, 3000–10,000 cm^−1^). The (meth)acrylate overtone peak at 6165 cm^−1^ was integrated for both spectra. DBC was calculated by the following equation:

$$DBC=\left(1-\left(\frac{A_{cured}}{A_{uncured}}\right)\right)*100$$

A_cured_ and A_uncured_ correspond to the integrated areas in the NIR spectrum (A_cured_: area of the (meth)acrylate peak for the cured material, A_uncured_: area of the (meth)acrylate peak for the uncured material).

#### 2.3.3. IvoBase High Impact

IvoBase high impact was cured using the injection-molding system IvoBase (Ivoclar Vivadent AG, Schaan, Liechtenstein) according to the manufacturer’s instructions. FS/FM and fracture toughness were measured according to ISO 20795-1:2013.

### 2.4. Scanning Transmission Electron Microscopy (STEM)

Samples of 70 nm thickness were prepared by ultramicrotomy (Leica model EM UC7, Wetzlar, Germany) at room temperature and deposited on the surface of high-resolution copper grids. In order to visualize the areas containing block copolymer, the sections were stained at 20 °C for 24 h with osmium tetroxide (OsO_4_) vapor. As the staining was found rather selective for the block copolymer, no further treatment of the sections was necessary. Observations were made using a JEOL ARM-200F (JEOL, Tokyo, Japan) transmission microscope operating at an accelerating voltage of 200 kV.

### 2.5. Scanning Electron Microscopy (SEM)

The fracture surfaces of the specimens were observed using a Zeiss Supra 40 VP SEM (Carl Zeiss AG, Oberkochen, Germany) at an accelerating voltage of 5 kV. The specimens were coated with gold/palladium (2 nm) before SEM observations.

## 3. Results

### 3.1. Difference Between 3D Printed and Bulk-Cured Materials

As a starting point for this work, a **DMA1**/OMIMA (1/1: wt/wt) formulation containing 5.0 wt% of **BCP1** and 3.0 wt% of SiO_2_-NPs was either photocured in bulk (in a mold, photocuring conditions: 20 mW cm^−2^ @ 405 nm and 100 mW cm^−2^ @ 450 nm, 10 min for each side) or 3D printed. The following 3D printing workflow was selected: The specimens were first 3D printed using a PrograPrint PR5 (Ivoclar Vivadent AG, Schaan, Liechtenstein, cleaned with wipes, and finally post-cured at RT in an LED cube 100 curing unit (Hönle, Gilching, Germany) using the same irradiation as for the bulk cured specimens (20 mW cm^−2^ @ 405 nm and 100 mW cm^−2^ @ 450 nm, 10 min for each side). Both the FS/FM and the fracture toughness were measured (Table 1). The results clearly showed that, under these conditions, the 3D-printed material exhibited significantly lower mechanical properties. FS, FM, K_max_ and W_f_ were far below the targeted ISO 20795-1:2013 values for materials with improved impact resistance (FS > 65 MPa, FM > 2000 MPa, K_max_ > 1.9 MPa m^1/2^ and W_f_ > 900 J m^−2^).

**Figure 2:**
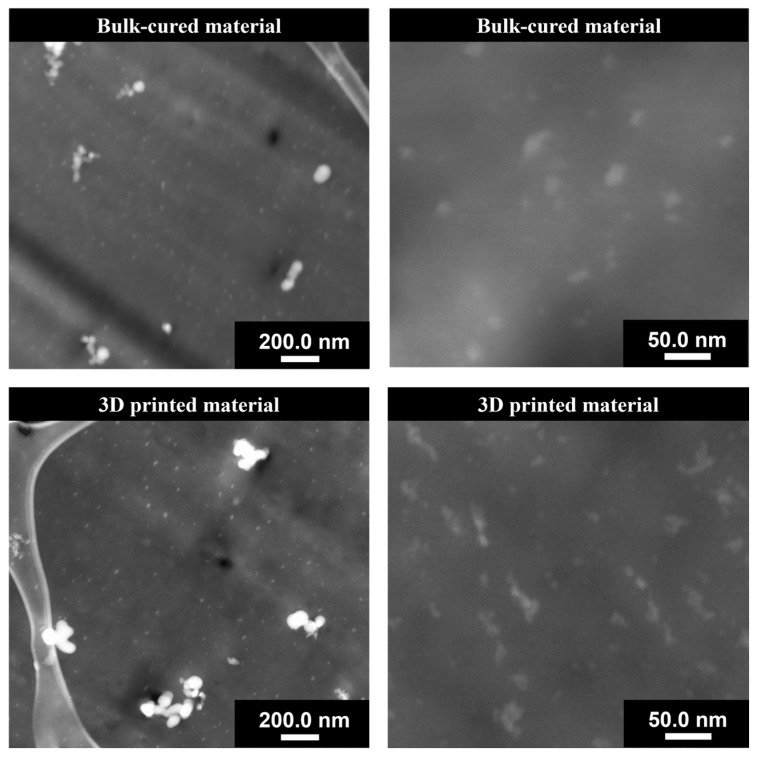
STEM micrographs of bulk-cured and 3D printed materials. Material: **DMA1**/OMIMA (1/1: wt/wt) + 5.0 wt% **BCP1** + 3.0 wt% SiO_2_-NPs.

STEM micrographs of both bulk-cured and printed specimens were acquired (Figure 2). The BCP nanostructures as well as SiO_2_-NPs can be clearly observed. BCP particles appear to be well dispersed in both materials. No clear difference could be noticed.

**Figure 3:**
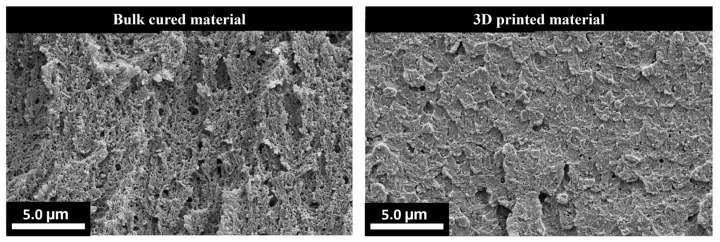
SEM micrographs of fracture surfaces for bulk-cured and 3D printed materials. Material: **DMA1**/OMIMA (1/1: wt/wt) + 5.0 wt% **BCP1** + 3.0 wt% SiO_2_-NPs.

SEM pictures of fracture surfaces were also taken (Figure 3). A rougher surface was observed for the bulk-cured material.

### 3.2. 3D Printing of BCP1 Containing Formulations: Influence of the Post-Curing Conditions on the Mechanical Properties

**Figure 4:**
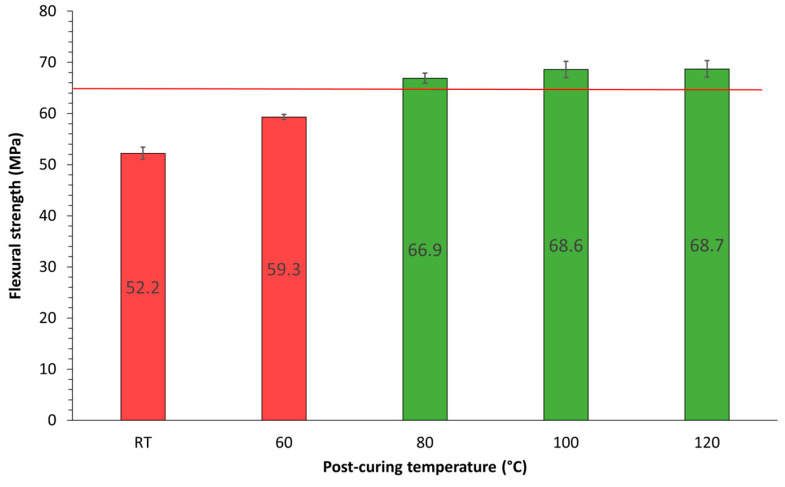
FS of printed materials that were post-cured at different temperatures. Material: **DMA1**/OMIMA (1/1: wt/wt) + 5.0 wt% **BCP1** + 3.0 wt% SiO_2_-NPs. The red line represents the minimum required value according to ISO 20795-1:2013 for type 4 (light-activated materials) denture base polymers.

**Figure 5:**
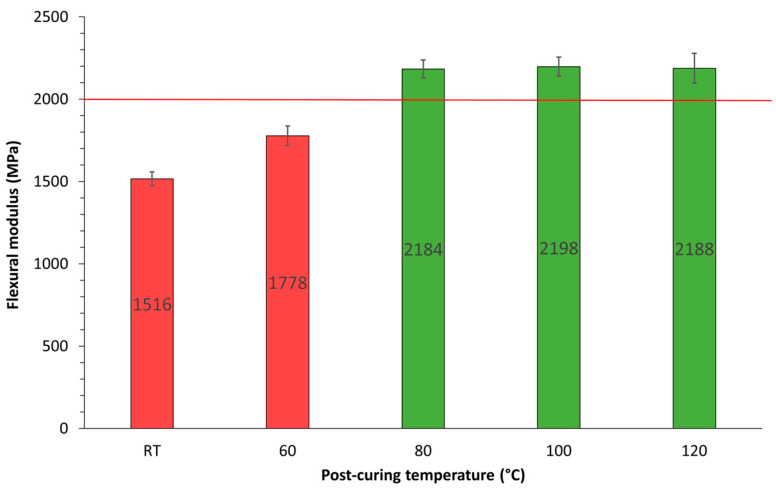
FM of printed materials that were post-cured at different temperatures. Material: **DMA1**/OMIMA (1/1: wt/wt) + 5.0 wt% + 3.0 wt% SiO_2_-NPs. The red line represents the minimum required value according to ISO 20795-1:2013 for type 4 (light-activated materials) denture base polymers.

**Figure 6:**
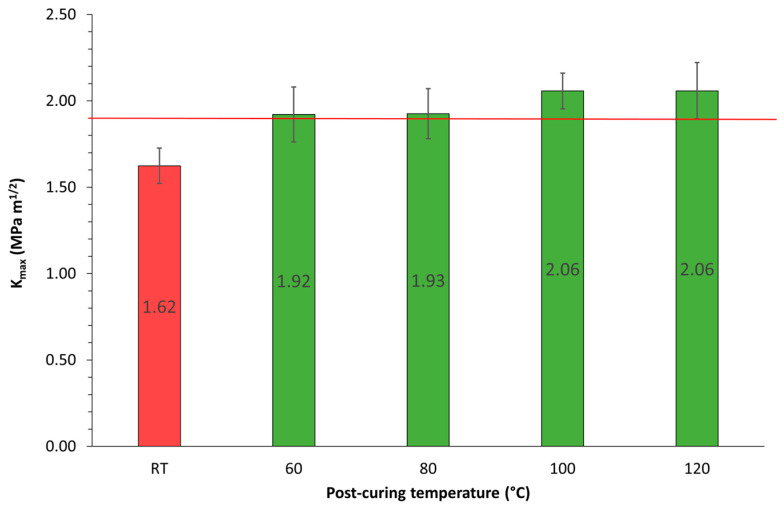
K_max_ of printed materials that were post-cured at different temperatures. Material: **DMA1**/OMIMA (1/1: wt/wt) + 5.0 wt% **BCP1** + 3.0 wt% SiO_2_-NPs. The red line represents the minimum required value according to ISO 20795-1:2013 for denture bases exhibiting improved impact resistance (high impact).

**Figure 7:**
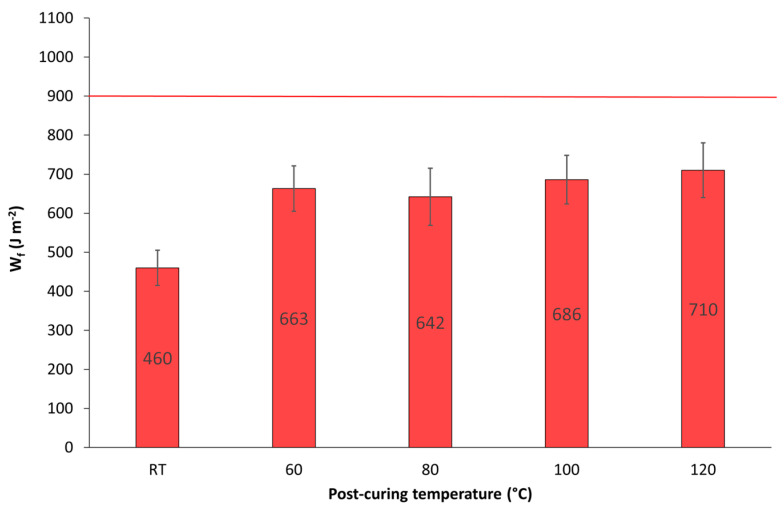
W_f_ of printed materials that were post-cured at different temperatures. Material: **DMA1**/OMIMA (1/1: wt/wt) + 5.0 wt% + 3.0 wt% SiO_2_-NPs. The red line represents the minimum required value according to ISO 20795-1:2013 for denture bases exhibiting improved impact resistance (high impact).

In order to improve the mechanical properties of 3D printed materials, the post-curing conditions were subsequently modified. Various temperatures were additionally selected for the post-processing step: 60 °C, 80 °C, 100 °C and 120 °C. FM, FS, K_max_ and W_f_ were assessed for each post-curing temperature (Figure 4, Figure 5, Figure 6 and Figure 7).

The results demonstrated that this parameter has a strong influence on the mechanical properties. Indeed, FS (RT: 52.2 ± 1.2 MPa; 60 °C: 59.3 ± 0.5 MPa) and FM (RT: 1516 ± 42 MPa; 60 °C: 1778 ± 59 MPa) obtained at RT and 60 °C were lower than the values measured at 80 °C (FS = 66.9 ± 1.0 MPa; FM = 2184 ± 54 MPa), 100 °C (FS = 68.6 ± 1.6 MPa; FM = 2198 ± 58 MPa), and 120 °C (FS = 68.7 ± 1.6 MPa; FM = 2188 ± 90 MPa). However, no difference was noticed between the specimens post-cured at 80 °C, 100 °C, or 120 °C. Using elevated temperatures during the post-processing step enabled the 3D printed **BCP1** based material to fulfill the ISO 20795-1:2013 requirements for type 4 (light-activated materials) denture base polymers in terms of FS (FS > 65 MPa) and FM (FM > 2000 MPa) and to reach similar values than for the bulk-cured material (FS = 62.7 ± 1.7 MPa; FM = 2028 ± 119 MPa; Table 1). Moreover, the post-curing temperature also significantly impacted the fracture toughness of the 3D-printed specimens. Indeed, a lower K_max_ value was obtained for the specimens post-cured at RT (K_max_ = 1.62 ± 0.10 MPa m^1/2^) compared to those that were post-cured at elevated temperatures (K_max_ > 1.92 MPa m^1/2^). Interestingly, regardless of the selected temperature (60 °C, 80 °C, 100 °C, or 120 °C), no significant differences were observed among the K_max_ values, all of which exceeded 1.9 MPa m^1/2^. Under these conditions, the ISO 20795-1:2013 requirements concerning materials with improved impact resistance were therefore met. A similar trend was evident for W_f_: Post-curing at RT produced a lower value (W_f_ = 460 ± 45 J m^−2^), whereas elevated temperatures yielded comparable and consistently higher W_f_ values (642 ± 73 J m^−2^ ≤ W_f_ ≤ 710 ± 70 J m^−2^). Unfortunately, for each tested post-curing temperature, the W_f_ was lower than the minimally required 900 J m^−2^ for high-impact denture bases (ISO 20795-1:2013) and did not reach the value obtained with the bulk-cured material.

The double-bond conversion (DBC) and T_g_ of printed materials were subsequently measured (Table 2). High DBC values (>95%) were reached for each post-curing condition. The RT post-processed material exhibited somewhat lower DBC. The DMTA results clearly showed that the higher the post-curing temperature, the higher the T_g_. The influence of the post-curing irradiation time on the mechanical properties of the 3D printed materials was also investigated. 3D printed specimens were post-cured at 100 °C during 2 × 5 min, 2 × 10 min and 2 × 20 min. Similar FS, FM, K_max_ and W_f_ values were obtained, independently of the post-curing time (Table 3).

### 3.3. 3D Printing of **BCP1** Containing Formulations: Influence of the **BCP1** and SiO_2_-NP Contents on the Mechanical Properties

**Figure 8:**
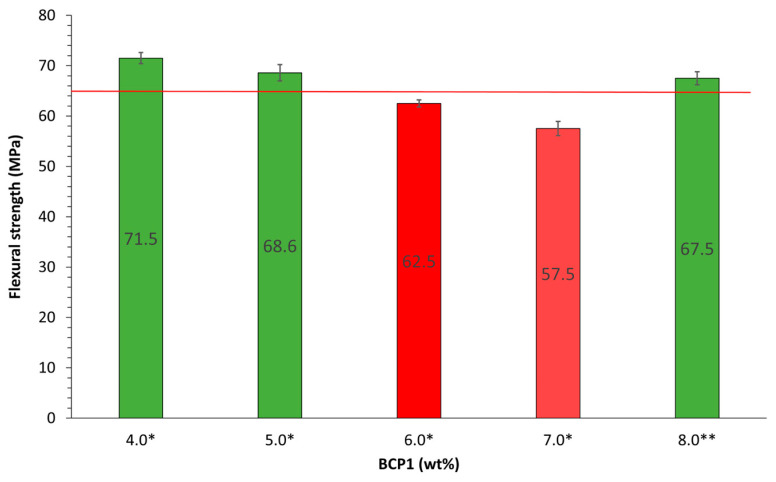
FS of printed **DMA1**/OMIMA (1/1: wt/wt) materials containing various amounts of **BCP1** and SiO_2_-NPs (*: 3.0 wt%; **: 10.0 wt%). The red line represents the minimum required value according to ISO 20795-1:2013 for type 4 (light-activated materials) denture base polymers.

**Figure 9:**
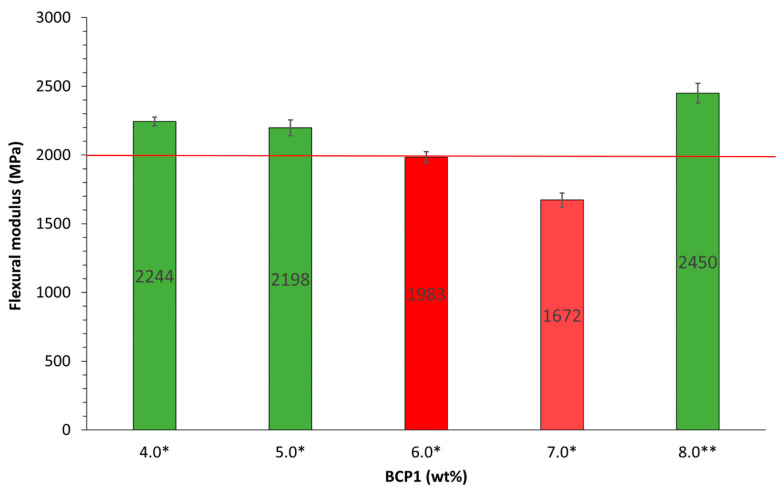
FM of printed **DMA1**/OMIMA (1/1: wt/wt) materials containing various amounts of **BCP1** and SiO_2_-NPs (*: 3.0 wt%; **: 10.0 wt%). The red line represents the minimum required value according to ISO 20795-1:2013 for type 4 (light-activated materials) denture base polymers.

**Figure 10:**
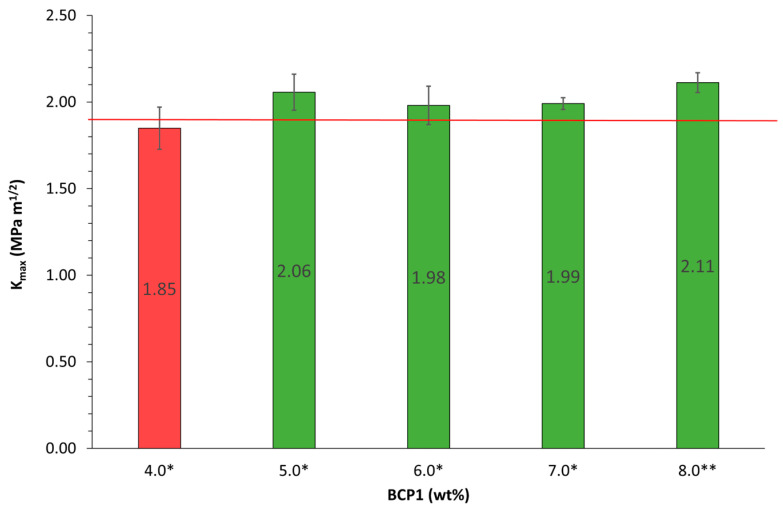
K_max_ of printed **DMA1**/OMIMA (1/1: wt/wt) materials containing various amounts of **BCP1** and SiO_2_-NPs (*: 3.0 wt%; **: 10.0 wt%). The red line represents the minimum required value according to ISO 20795-1:2013 for denture bases exhibiting improved impact resistance (high impact).

**Figure 11:**
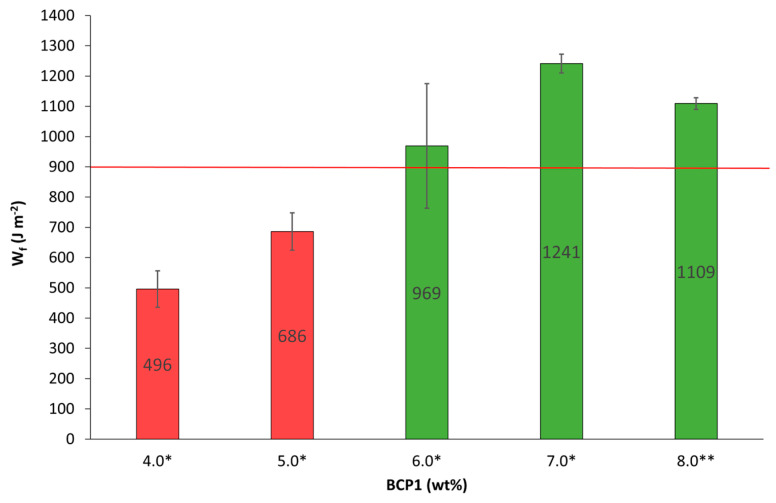
W_f_ of printed **DMA1**/OMIMA (1/1: wt/wt) materials containing various amounts of **BCP1** and SiO_2_-NPs (*: 3.0 wt%; **: 10.0 wt%). The red line represents the minimum required value according to ISO 20795-1:2013 for denture bases exhibiting improved impact resistance (high impact).

The influence of the **BCP1** content on both the FS/FM and the fracture toughness of DLP-printed materials was studied (Figure 8, Figure 9, Figure 10 and Figure 11). **DMA1**/OMIMA (1/1: wt/wt) formulations containing 4.0 wt%, 6.0 wt%, and 7.0 wt% of **BCP1** were first selected (each formulation additionally contained 3.0 wt% SiO_2_-NPs).

The following trends can clearly be identified: The higher the **BCP1** content, the lower the FS and FM values. On the other hand, W_f_ increases together with the **BCP1** amounts. A **BCP1** content of at least 6.0 wt% was needed to reach the minimum requirements for high impact in terms of K_max_ and W_f_ (ISO 20795-1:2013). However, FS and FM were slightly below the targeted values (FS ≤ 62.5 ± 0.7 MPa; FM ≤ 1983 ± 40 MPa). A formulation containing 8.0 wt% **BCP1** and having an increased SiO_2_-NPs content of 10.0 wt% was finally prepared. This resin was able to fulfill all requirements for materials with improved impact resistance (FS = 67.5 ± 1.3 MPa, FM = 2450 ± 71 MPa, K_max_ = 2.11 ± 0.06 MPa m^1/2^, W_f_ = 1109 ± 19 J m^−2^).

### 3.4. Comparison with the State of the Art

Two commercially available 3D printing denture base materials were also evaluated. Moreover, IvoBase high impact was selected as a PMMA-based reference material. FS, FM as well as K_max_ and W_f_ were measured (Table 4).

Printodent^®^ GR-14.2 denture HI exhibited satisfactory FS and FM values (FS = 69.2 ± 1.8 MPa, FM = 2153 ± 76 MPa). However, the printed material was brittle and presented extremely low K_max_ and W_f_ values (K_max_ = 0.82 ± 0.04 MPa m^1/2^, W_f_ = 79 ± 10 J m^−2^). On the other hand, a high fracture toughness was obtained using Lucitone Digital Print^TM^ 3D denture base (K_max_ = 1.92 ± 0.09 MPa m^1/2^, W_f_ = 1272 ± 177 J m^−2^). However, the measured FS (FS = 56.7 ± 1.9 MPa) was significantly lower than the required 65 MPa (ISO20795-1:2013). Interestingly, the mechanical properties measured with the optimized **DMA1**/OMIMA formulation (8.0 wt% **BCP1**; 10.0 wt% SiO_2_-NPs content) and with the PMMA based material (IvoBase high impact) were in a similar range.

## 4. Discussion

The objective of this work was to transfer the BCP toughening technology to DLP 3D printing. A resin formulation based on a **DMA1**/OMIMA/**BCP1** combination was selected (with TPO as photoinitiator) for this study. Our research group recently showed that this formulation was suitable for the development of light-cured high-impact denture bases, due to the excellent toughening ability of **BCP1** [33]. However, these materials were photocured in a mold, raising the question of whether this technology can be effectively transferred to DLP 3D printing.

To answer this question, the **BCP1** containing formulation (**DMA1**/OMIMA (1/1: wt/wt) + 5.0 wt% **BCP1** + 3.0 wt% SiO_2_-NPs) was printed using a standard workflow. A PrograPrint PR5 was selected as the DLP printer. Following printing, specimens were post-cured in an LED cube 100 curing unit at RT (20 mW cm^−2^ @ 405 nm and 100 mW cm^−2^ @ 450 nm, 10 min from both sides). The mechanical properties (FS/FM and fracture toughness) were measured and compared with the values of the bulk-cured material (Table 1). A significant drop in the mechanical properties was observed when the material was 3D printed, to values far below the minimum targeted requirements for high-impact denture bases. This strong decrease cannot be due to different nanomorphologies. Indeed, STEM micrographs demonstrated that similar nanostructures were observed for 3D-printed and bulk-cured materials (Figure 2). It was actually expected, as the well-dispersed nano-objects are formed via self-assembly of **BCP1** in the uncured **DMA1**/OMIMA mixture [33]. The lower mechanical properties of the printed material could, however, partially arise from the incomplete conversion after post-curing (DBC = 95 ± 1%, Table 2). Interestingly, an increased roughness was observed on the SEM picture of the fracture surface corresponding to the bulk-cured material (Figure 3). This observation clearly correlates with the K_max_ and W_f_ values.

In order to improve the mechanical properties of 3D printed **BCP1** based materials, we subsequently decided to focus on the variation in the post-curing conditions. The post-curing temperature has recently been shown to play a key role in the physical properties of printed materials [34,35,36,37]. As an example, K. Qi et al. printed a dimethacrylate-based nanocomposite using SLA technology and evaluated the post-curing at either 40 °C or 80 °C [35]. The specimens that were post-cured at 80 °C exhibited significantly higher FS and FM. Therefore, different temperatures (60 °C, 80 °C, 100 °C, and 120 °C) were selected for the post-curing step in the present work. The results clearly demonstrated that the post-curing temperature has a strong influence on FS and FM (Figure 4 and Figure 5). Materials post-cured above 80 °C exhibited FS and FM values exceeding the minimum requirements for type 4 (light-activated materials) denture base polymers (ISO 20795-1:2013). These results are therefore in agreement with the conclusions of K. Qi et al. [35]. It is worth mentioning that both the FS and FM of these printed materials were similar to the bulk cured specimens. The same trend was observed for the fracture toughness (Figure 6 and Figure 7). Both K_max_ and W_f_ values were strongly improved upon heating during the post-curing step. Unfortunately, and independently of the selected post-curing temperature, the measured W_f_ remained significantly below the 900 J m^−2^ threshold required for high impact denture bases. This outcome might suggest that the network structure of printed versus bulk-cured materials is different (different crosslinking density). It might also be due to the presence of layers in the printed material or to the different photocuring process (in two steps for printed materials: Irradiation during printing, followed by a second irradiation in a post-curing unit). Nonetheless, it must be highlighted that the obtained fracture toughness is significantly improved in comparison to conventional denture bases. As an example, ProBase Hot (heat-cure PMMA-based conventional denture base material from Ivoclar Vivadent AG) exhibits a maximum stress intensity factor (K_max_) of 1.44 ± 0.18 MPa m^1/2^ and a total work of fracture (W_f_) of 270 ± 30 J m^−2^ [38].

The post-curing temperature was also shown to have a strong influence on DBC and T_g_ of printed materials (Table 2). Heating during the post-curing step led to an increase in these values, which might explain the trend observed with regard to the values obtained for FS and FM. The T_g_ of the fully cured materials is slightly below 80 °C. With post-curing the material above the T_g_, improvement in the polymer chain mobility is created, resulting in higher DBC, FS and FM. 100 °C seems to be the most suitable post-curing temperature, as no further advantages were observed at 120 °C. Using these conditions, the irradiation time was varied. The results showed that this parameter did not have any influence on the mechanical properties of printed materials (Table 3).

Moreover, it is well known that the amount of BCP strongly influences the fracture toughness as well as FS/FM of bulk light-cured materials [33]. In this contribution, **DMA1**/OMIMA (1/1: wt/wt) formulations containing 4.0 wt%, 6.0 wt%, and 7.0 wt% of **BCP1** were also 3D printed and evaluated. As expected, higher **BCP1** content negatively influenced both FS and FM (Figure 8 and Figure 9). On the other hand, the higher the amounts of **BCP1**, the higher the W_f_. An addition of 6.0 wt% of **BCP1** was sufficient to exceed the minimum required K_max_ and W_f_ values for high-impact denture bases (Figure 10 and Figure 11). Unfortunately, FS and FM were slightly below the aimed values. In order to further optimize the formulation, both the **BCP1** (8.0 wt%) and the SiO_2_-NP (10.0 wt%) contents were increased. The addition of higher inorganic filler amounts was expected to improve FM. Indeed, SiO_2_-NPs inherently have high FM. Consequently, their incorporation into a softer matrix typically results in an increase in FM values. This strategy proved effective, as the new resin was able to fulfill all criteria (FS, FM, K_max_ and W_f_) for materials with improved impact resistance (ISO 20795-1:2013). Two commercially available 3D printing denture base materials (Printodent^®^ GR-14.2 denture HI and Lucitone Digital Print^TM^ 3D denture base) were additionally selected and evaluated (Table 4). Printodent^®^ GR-14.2 denture HI was found to be extremely brittle and therefore rather unsuitable for denture bases. According to the state of the art, Lucitone Digital Print^TM^ 3D denture base is currently the only 3D printing material that exhibits high fracture toughness. Our results confirm this claim, as both K_max_ and W_f_ were above the minimum required values for high impact materials (ISO 20795-1:2013). Moreover, our measurements confirm the observations of Lawson et al. and Coldea et al.: FS remains significantly lower than the aimed 65 MPa [18,19]. In this context, the optimized **BCP1**-containing resin is the only one able to fully meet the ISO20795-1:2013 requirements and can therefore be considered as a 3D printing high-impact denture base. Even if the PMMA-based gold standard material (IvoBase high impact) still exhibits higher FS and W_f_ values, the differences are modest (Table 4). In the near future, 3D-printed denture bases should be able to exhibit similar mechanical properties to PMMA-based reference materials.

## 5. Conclusions

In this contribution, **DMA1**/OMIMA containing various amounts of **BCP1** were successfully printed using DLP technology. Interestingly, it was shown that the transfer of bulk photocuring (in molds) to 3D printing led to a decrease in fracture toughness. Although the addition of **BCP1** clearly led to a strong increase in K_max_ and W_f_ of 3D-printed materials, the improvement was not as strong as for bulk-cured resins. This study also clearly highlighted the impact of the post-curing temperature on the 3D-printed materials’ mechanical properties. It seems to be essential to heat the printed material above T_g_ during the post-processing step in order to obtain the best properties. Using an optimized formulation and an improved 3D printing workflow, it was possible to fulfill all the requirements for denture bases with improved impact resistance (ISO 20795-1:2013). This work therefore clearly demonstrates the high potential of the BCP toughening technology for the development of 3D printing high-impact denture bases that would exhibit similar mechanical properties to the gold standard PMMA based materials.

## Figures and Tables

**Table 1:** FS/FM, K_max_ and W_f_ of bulk-cured and 3D printed (post-curing at RT) **DMA1**/OMIMA (1/1: wt/wt) materials containing 5.0 wt% of **BCP1** and 3.0 wt% of SiO_2_-NPs.

Curing Conditions	FS ISO 50 h 37 °C (MPa)	FM ISO 50 h 37 °C (MPa)	K_max_ (MPa m^1/2^)	W_f_ (J m^−2^)
Bulk curing	62.7 ± 1.7	2028 ± 119	2.25 ± 0.05	1439 ± 31
3D printing	52.2 ± 1.2	1516 ± 42	1.62 ± 0.10	460 ± 45

**Table 2:** T_g_ and DBC of printed materials that were post-cured at different temperatures. Material: **DMA1**/OMIMA (1/1: wt/wt) + 5.0 wt% **BCP1** + 3.0 wt% SiO_2_-NPs.

Post-Curing Temperature (°C)	DBC (%)	T_g_ (°C)
RT	95 ± 1	65.5
60	98 ± 1	70.0
80	99 ± 1	73.0
100	100	74.0
120	100	76.2

**Table 3:** FS, FM, K_max_, and W_f_ measured after various post-curing irradiation times at 100 °C. Material: **DMA1**/OMIMA (1/1: wt/wt) + 5.0 wt% **BCP1** + 3.0 wt% SiO_2_-NPs.

Post-Curing Irradiation Time (min)	FS ISO 50 h 37 °C (MPa)	FM ISO 50 h 37 °C (MPa)	K_max_ (MPa m^1/2^)	W_f_ (J m^−2^)
2 × 5	67.0 ± 1.4	2135 ± 47	2.07 ± 0.17	677 ± 80
2 × 10	68.6 ± 1.6	2198 ± 58	2.06 ± 0.10	686 ± 62
2 × 20	69.2 ± 0.6	2145 ± 38	2.02 ± 0.11	615 ± 64

**Table 4:** FS, FM, K_max_, and W_f_ of three commercially available materials.

Material	FS ISO 50 h 37 °C (MPa)	FM ISO 50 h 37 °C (MPa)	K_max_ (MPa m^1/2^)	W_f_ (J m^−2^)
Printodent^®^ GR-14.2 denture HI	69.2 ± 1.8	2153 ± 76	0.82 ± 0.04	79 ± 10
Lucitone Digital Print^TM^ 3D denture base	56.7 ± 1.9	2144 ± 121	1.92 ± 0.09	1272 ± 177
IvoBase high impact	73.8 ± 2.0	2361 ± 53	2.12 ± 0.07	1366 ± 72

## Data Availability

The data presented in this study are available on request from the corresponding author.
